# A scalable MapReduce-based design of an unsupervised entity resolution system

**DOI:** 10.3389/fdata.2024.1296552

**Published:** 2024-03-01

**Authors:** Nicholas Kofi Akortia Hagan, John R. Talburt, Kris E. Anderson, Deasia Hagan

**Affiliations:** ^1^Department of Information Sciences, University of Arkansas at Little Rock, Little Rock, AR, United States; ^2^Department of Rhetoric and Writing, University of Arkansas at Little Rock, Little Rock, AR, United States

**Keywords:** data curation, entity resolution, Data Washing Machine, MapReduce, Hadoop Distributed File System

## Abstract

Traditional data curation processes typically depend on human intervention. As data volume and variety grow exponentially, organizations are striving to increase efficiency of their data processes by automating manual processes and making them as unsupervised as possible. An additional challenge is to make these unsupervised processes scalable to meet the demands of increased data volume. This paper describes the parallelization of an unsupervised entity resolution (ER) process. ER is a component of many different data curation processes because it clusters records from multiple data sources that refer to the same real-world entity, such as the same customer, patient, or product. The ability to scale ER processes is particularly important because the computation effort of ER increases quadratically with data volume. The Data Washing Machine (DWM) is an already proposed unsupervised ER system which clusters references from diverse data sources. This work aims at solving the single-threaded nature of the DWM by adopting the parallelization nature of Hadoop MapReduce. However, the proposed parallelization method can be applied to both supervised systems, where matching rules are created by experts, and unsupervised systems, where expert intervention is not required. The DWM uses an entropy measure to self-evaluate the quality of record clustering. The current single-threaded implementations of the DWM in Python and Java are not scalable beyond a few 1,000 records and rely on large, shared memory. The objective of this research is to solve the major two shortcomings of the current design of the DWM which are the creation and usage of shared memory and lack of scalability by leveraging on the power of Hadoop MapReduce. We propose Hadoop Data Washing Machine (HDWM), a MapReduce implementation of the legacy DWM. The scalability of the proposed system is displayed using publicly available ER datasets. Based on results from our experiment, we conclude that HDWM can cluster from 1,000's to millions of equivalent references using multiple computational nodes with independent RAM and CPU cores.

## Introduction

Data curation is simply the management of data throughout its life cycle, from planning and acquisition through data standardization, integration, and productization to archiving and disposal. This process is often time-consuming because it requires a lot of human supervision. As the size of data has grown, the need to remove human-in-the-loop from the data curation process has become increasingly important to save time and reduce costs.

With the advent of big data systems, there is now a shift from relational database management systems to non-relational technologies such as Hive and MongoDB, and distributed processing technologies such as Hadoop MapReduce and PySpark. This requires organizations to re-engineer many of their data curation processes to incorporate big data technologies to remain competitive.

Entity resolution (ER) is a data curation process which decides whether computer references to two real-world objects are referring to the same entity or different entities (Talburt and Zhou, 2015). Entities here refer to real-world objects having distinct identities, such as people (customers, patients, students, etc.), products, and locations. If two references refer to the same entity, they are said to be equivalent references. The goal of an ER process is 2-fold. The first goal is to create clusters where each cluster contains only references to one particular entity (equivalent references). The second is that all references to a particular entity are in the same cluster. The measure of achievement for the first goal is the precision of the ER process, and the achievement measure for the second goal is the recall of the ER process.

ER is foundational to many data curation processes, such as master data management (MDM) and data integration or data fusion (Talburt et al., 2019). According to Kolb et al. (2012a), MapReduce is a great fit for ER processing because each comparison between a pair of entity references is independent in nature and, therefore, can be carried out in parallel.

However, the challenge is that many traditional ER processes rely on in-memory access to information across all input references, a requirement that goes against the distributed computing paradigm. Consequently, most traditional ER systems must be refactored to remove this dependency before they can be implemented efficiently in MapReduce, Spark, and other distributed processing systems.

The DWM, a concept first introduced by Al Sarkhi and Talburt (2018, 2019) is a system designed for unsupervised ER (Talburt et al., 2020). The DWM represents the movement toward the development of unsupervised data curation processes (Talburt et al., 2023). It reverses the traditional ER paradigm of starting by cleaning and standardizing the data sources and then matching and linking. Instead, it attempts to first link pairs of references to the same entity, cluster the references, and then use the clusters to clean and standardize the data. As the DWM clusters references, it self-evaluates the quality of each cluster to decide whether the cluster should be kept or if the references in the cluster should be sent back for re-linking using a higher matching threshold. This allows the DWM to run iteratively without supervision.

The HDWM uses the same 28 parameter settings to cluster and clean equivalent references. A total of 14 out of the 28 parameters are set using an automated Parameter Discovery Process (PDP). The process of determining which parameter values to use for a particular dataset is found by an automated Parameter Discovery Process (PDP). The PDP process is executed prior to running the DWM (Anderson et al., 2023). The PDP gives the user a set of optimal starting parameter values using a combination of input data statistics, historical settings, regression analysis, and entropy-aided grid search. Most of the statistics relate to tokens in input data, such as the average number of tokens per reference, the count of unique tokens, the average token frequency, and the ratio of numeric to non-numeric tokens.

The input for the DWM is a merged file of data sets from multiple sources. The different inputs may have different formats and metadata alignments. The only assumptions are,

- Each reference has a unique record identifier,
- All references refer to a common set of entities,
- The records only contain entity identity information.

No data preprocessing is performed on these files prior to the entity resolution task. A tokenization process is applied to the merged data set by first removing any column headers, if any, and applying one of the tokenization methods. The output of the tokenization process is a dictionary of tokens and token frequencies, which serve as a central source from which other processes depend. The DWM uses a frequency-based blocking approach. A parameter value called beta is used to create such blocks where all references sharing a token with a frequency between 2 and the beta value form a block. For instance, if beta is 4, references sharing a token with frequency 2, 3, and 4 form a block. References in the same block are then compared for similarity by first removing any stopwords from each reference. Stopwords are tokens with a frequency higher than a sigma threshold. References that are similar are then linked using another parameter called mu. Mu is a similarity threshold where similar pairs of references with a similarity score above the mu value are linked. The linked pairs are then clustered using a transitive closure process. The quality of the clusters is then evaluated using an entropy-based measure. Another DWM parameter called epsilon sets the quality threshold. If a cluster scores above epsilon, it is kept as a good cluster. If a cluster scores below epsilon, the references are sent back for re-blocking and re-linking based on an incrementally higher value of mu.

The current design of the DWM is single-threaded and linear in nature, limiting it to processing only a few 1,000 references. Also, the DWM creates and uses a shared dictionary of tokens, which results in an “out-of-memory” problem as the size of data increases and the core memory is not large enough to hold the dictionary.

The goal of this research is to demonstrate how the DWM, and other ER systems can be refactored into a distributed computing environment for scalability. This research uses Hadoop MapReduce to carry out the basic processes used in the single-threaded DWM while following the requirements of distributed processing. The most challenging requirements are to avoid a large, shared memory space and the need for any one processor to directly communicate or exchange data with another processor in real time.

Hadoop^1^ is a software library from the Apache Open-Source Software Foundation. It was originally developed by Google to index the words on all internet website pages. Instead of using a large supercomputer, MapReduce (Dean and Ghemawat, 2008) and other distributed operating systems rely on a network of many small commodity computers to do the work. Google later made the system open source through the Apache Foundation. Newer distributed processing systems such as Spark are built on top of MapReduce processes. Hadoop is popular for its fault tolerance capability and the ability to easily scale up from a single computer to a cluster of hundreds or even 1,000's of smaller computers working simultaneously. The main architecture of Hadoop includes Hadoop Common, which is the basic utility upon which other modules are built; Hadoop Distributed File System (HDFS) which is the distributed file system used as the primary source of data storage for any Hadoop application; Hadoop Yarn which serves as a framework used by Hadoop for job scheduling and resource management in a cluster for all computational nodes; and Hadoop MapReduce which is the processing engine of Hadoop.

This research makes the following contributions:

- We introduce the Hadoop Data Washing Machine, a MapReduce implementation of an already existing proof-of-concept unsupervised ER system, DWM. We capitalize on the parallel nature of MapReduce to solve the single-threaded design nature of the DWM.
- We present a refactoring method that can be applied to other single memory ER systems into distributed environment using Hadoop MapReduce. The usage of our proposed methods will help to get away from the single memory space which is common in most traditional ER systems.
- We benchmark HDWM with the legacy DWM using 18 generated data samples and demonstrate that HDWM obtains the same clustering results. This is an indication that all the necessary steps in the DWM have been completely refactored into Hadoop MapReduce.
- We demonstrate the scalability of HDWM using up to 50 million publicly available benchmark entity clustering datasets.

## Related work

Since the advent of big data, many attempts have been made to use distributed computing to scale data-intensive systems in diversified fields and industries including entity resolution.

A scalable approach was adopted to redesign OYSTER, a supervised ER system for clustering equivalent references (Al Sarkhi and Talburt, 2020). This work uses frequency-based blocking and stop word removal. The scalable implementation adopted in this research involved two main preprocessing stages. First, the frequency of each of the tokens is calculated, and second, all excluded blocking tokens and stopwords are eliminated, leaving a skinny reference pair which will be compared for similarity using the scoring matrix. The work uses normalized Levenshtein Edit Distance (nLED) to compare the pair of references that come out of the block deduplication phase.

The DWM has birthed many advancements since its introduction. One of the major areas of advancement is a graph-based implementation of the system. ModER, as proposed in Ebeid et al. (2022), eliminates the iterative nature in the processing pipeline of the DWM by further dividing the recast graph into smaller graphs and finding each sub-graph's connected components using a transitive closure logic (Seidl et al., 2012; Kolb et al., 2014). ModER also eliminates the need for a user setting similarity and cluster quality thresholds by using modularity as a cluster quality matrix.

One critical area of research was developing an unsupervised mechanism for estimating the optimal parameters to be used in the DWM. Prior work had been done in Al Sarkhi and Talburt (2018, 2019) for a matrix comparator used for linking equivalent references. However, the matrix comparator is just a piece of the puzzle, and further works was needed for the entire DWM to estimate the best starting values for all 28 parameters used by the system. This birthed the Parameter Discovery Process (Anderson et al., 2023). The PDP uses statistics from the input data and logistic regression mechanism to compute the best starting values for each of the parameters used in the DWM.

Dedoop is a user-friendly MapReduce-based engine that translates user-defined ER settings into MapReduce jobs (Kolb et al., 2012a). It implements several blocking processes, similarity computation between references, and matching similar references, which are all translated into individual MapReduce processes. Dedoop performs blocking in a mapping phase and similarity computation in a reducer program. It also has a set of supervised machine learning libraries used to find and compare reference similarities.

Several blocking and indexing techniques (Christen, 2012) have been proposed for ER, and the usage of a single blocking key is a dominant approach in finding pairs of references that are potential matches (Papadakis et al., 2020). However, certain pairs that are supposed to be matched do not get matched because such pairs did not end up in the same block, hence reducing the number of complete pairs. A multi-key approach to block references for ER, ensuring every record is accounted for at the expense of having ununiform blocks and having the same record appearing in multiple blocks has also been proposed by Mittal et al. (2022). This work, however, does not account for the need to perform cluster-level comparisons using transitive closure, which is an inevitable step in forming clusters of equivalent references. Although the usage of multiple blocking keys has proven to be a promising approach to identify duplicated entities which need to be compared and matched, it undoubtedly generates more duplicate pairs for comparison.

Nascimento et al. (2020a) proposed a blocking pruning model to reduce the duplicate pairs formed and control the size of blocks when using multiple blocking keys approach. The proposed approach uses the shrink, split, merge, and exclude operations to ensure only blocks that are highly probable to match are send for comparison. The algorithm takes as input, the dataset in question and a set of indexing or blocking rules, then the input data is shrunk by only focusing on blocks that will produce matching entities. After this step, it is possible that there will be unbalanced blocks and so the next step is to split larger blocks into much smaller blocks which are then merged with already smaller blocks created from the previous step. All blocks that are unlikely to match are finally excluded.

Real-world data are inevitably characterized by several useful embedded information that could be used in any application. Simonini et al. (2016) proposed Blast and illustrates how these loose schema statistics from the data could be utilized to enhance blocks in entity resolution. Loose schema is extracted using the similarity calculation for each attribute in the dataset. They used token-blocking and graph-based meta-blocking approach to group references having similar keys. In contrast, our approach uses a loose schema information from each reference such as the frequency of a particular token within the entire dataset and then a frequency-based blocking approach is utilized to group all references having the same blocking key.

To reduce the computational complexity caused by comparing each record to every other records even further, there is the need to avoid redundant pairs that are formed from blocks. In a MapReduce-based entity resolution, there is the possibility of having references with the same identifier end up in the same computational node because they share the same blocking key. Another phase in the entity resolution process that could be affected by redundancy is the transitivity phase where matches are further clustered. To solve these problems, Yan et al. (2015) proposed “multi-sig-er” method for parallel entity resolution using a two-phased approach, filtration and verification. The filtration phase assumes that a pair of similar references must have one or more common signature. This is the first level of redundant elimination where all dissimilar pairs are ignored. The redundancy elimination occurs in a reducer program where similar pairs share the same key. The candidate pairs are then verified using transitivity logic of further clustering blocks before comparing pairs for similarity and linking. Although this approach has proven to be a working approach, it does not support incremental data processing.

Kolb et al. (2013) proposed a deterministic approach where each reducer will choose a single signature from a set of signatures associated with a pair of reference. The algorithm considers the smallest possible key in a block which ends up in a reducer, and then eliminates all other pairs whose key is high in the block. In the mapper phase an emitted key-value pair is accompanied by a set of signatures. The reducer further analyzes each pair for determine if a set has no signature in common (disjoint set of signatures), such pairs are eliminated. If the pair have a common signature, they are kept and compared.

Kolb et al. (2011) presented a way of merging machine learning and MapReduce to solve the computational complexity problem in entity resolution. Entity resolution as a process can be categorized as a classification problem where pairs of references can either be matched or unmatched pairs. Although their approach reduces the execution time of learning-based ER, it does not implement blocking.

To effectively achieve the required reduction in computational time of MapReduce-based ER, there is a need for an even distribution of data across mapper and reducer tasks. This has made load-balancing an inevitable technique in using MapReduce for ER. Kolb et al. (2012b) proposed two types of load-balancing techniques, BlockSplit and PairRange, using MapReduce. Before any of these two strategies are used, a block preprocessing step is performed to form a block distribution matrix in the mapper phase, and then the load-balancing is performed in the reducer phase. BlockSplit uses block size and ensures larger blocks are further partitioned into smaller sub-blocks, which are then fed into the reducer. PairRange number-tags pairs and ensures equal comparisons across blocks.

Jin et al. (2017) processed MrEm which aims to ensure load-balancing and avoid redundant matching using multiple blocking techniques. They provide a four-phased approach to solving the load-imbalance problem and pair redundancy problems simultaneously, a solution missing from previous works. In their approach, all blocks are further divided into smaller sub-blocks and are assigned an identical key. All sub-blocks having the same key then end up in the same reducer to identify and eliminate duplicates. They provide two types of block sub-block, the self-join sub-block which comprise of all records in each sub-group and the cross-join sub-block which comprise of intersecting records from two sub-groups. MrEm performs data shuffling within blocks rather than within the entire dataset. The idea of block-based shuffling is critical and helps to reduce the I/O overhead after a mapper and before a reducer job begins.

In MapReduce applications, map tasks and reduce tasks run in isolation, and there is no shared memory between the computational nodes in the cluster. These two capabilities in MapReduce pose a challenge for regression unit testing in the software development lifecycle. The work of Pullen et al. (2018) highlights a mechanism to test the performance of MapReduce-based ER engines using regression analysis. This framework was developed for the High-Performance Entity Resolution (HiPER) system but can be adopted in different domains and applications. The HiPER Testing Framework (HTF) compares its results to a set of expected results and determines whether the software test was a pass or failure.

The exponential growth in data has given need for organizations to measure the computational cost of deploying data quality algorithms on platforms that give the best return on investment. Data Quality Service-Level Agreement (DQSLA) is a contract between a service and its customers, and it includes specifications on execution time of data quality algorithms, time restriction for these algorithms, as well as the penalties that may be applied to the service. This can be a costly endeavor for either party in the contract if any of the terms of conditions is not met. To help alleviate these issues, Nascimento et al. (2020b) proposed a theoretical model for estimating the execution cost of record linkage algorithms in a cloud computing environment. In their work, they quantified the cost of execution as the summation of indexing/blocking, the similarity comparison phase of the process, and the classification of two entities as either match or non-match. According to the authors, these values are influenced by the employed algorithm, and the cloud computing environment employed. Although the model works best certain use-cases, it does not consider the time taken by transitive closure algorithm which are employed in the execution process. Transitive closure is an inevitable phase in the ER process and record clustering.

## Methodology

The design approach of HDWM^2^ is a complete refactor of the traditional DWM by mimicking the main processes of the legacy DWM using Hadoop MapReduce (Dean and Ghemawat, 2008). MapReduce is a parallel programming module used for processing larger data sets at a faster pace, and the programming is expressed in two main functions, mapper, and reducer. In Equation 1, the mapper function takes a key-value pair as an input and produces an intermediate key-value pair, which serves as input for the reducer function that emits a final key-value pair as shown in Equation 2. The reducer shuffles and sorts a mapper's output based on key groups and aggregates all values belonging to the same key group.


(1) $$\begin{array}{l} Mapper:\;\left(key_{in}\;,\;value_{out}\right)\;\to list\left(key_{tmp}\;,\;value_{tmp}\right) \end{array}$$

(2) $$\begin{array}{l} Reducer:\;\left(key_{tmp}\;,\;list\left(value_{out}\right)\right)\;\to list\left(key_{out}\;,\;value_{out}\right) \end{array}$$

**Figure 1:**
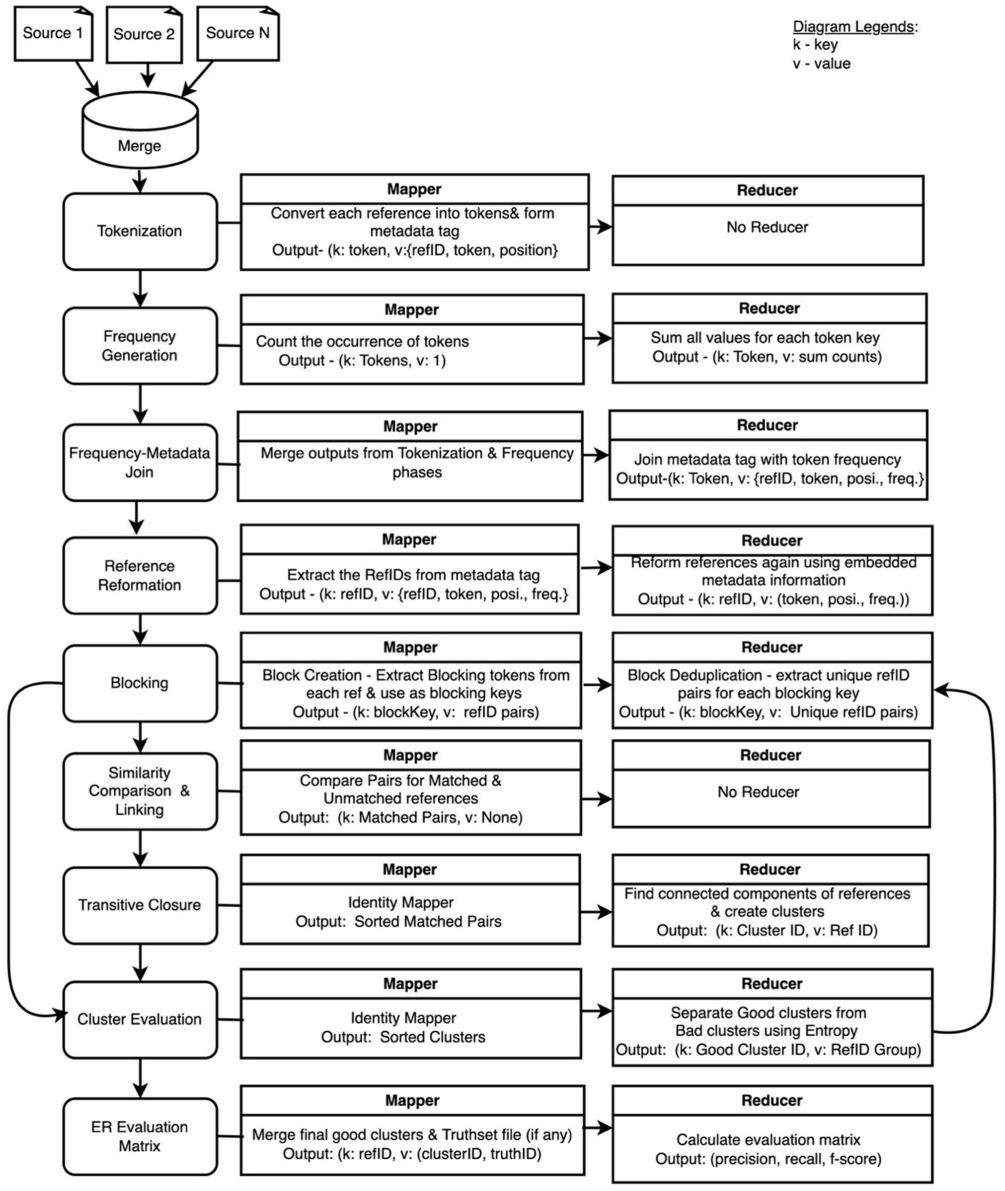
Hadoop Data Washing Machine design architecture using MapReduce.

Figure 1 shows a set of refactoring steps involved in the development of the proposed HDWM. Each of the processes uses a mapper and reducer function written in Python. Although MapReduce was originally written in Java, the Hadoop Streaming API allows non-Java programmers to easily write MapReduce applications in any programming language of their choice by using system standard input and output to read and write data. The Hadoop Streaming API was extensively utilized to write the mapper and reducer functions in HDWM.

### Tokenization

The tokenization process reads the merged data set and removes all non-word characters and the column header row if column headers are present. The column header is eliminated in the spirit of unsupervised data curation not utilizing headers for clustering references. Each row of record is then broken down into individual tokens. The output of this process is in the form of tokens as key and value in the form of a metadata tag containing the reference identifier where the token came from, the position of the token, and the token itself. HDWM uses only intrinsic metadata from the given data set for form a tag during the tokenization process. The tokenization process also uses one of two main tokenization algorithms, tokenizer splitter, and tokenizer compress. The splitter function splits each reference into individual tokens using the given delimiter. The opposite is true for the compress function. The tokenization process occurs at the mapping phase and statistics such as total tokens found, total numeric tokens, unique token count, and duplicate tokens are all extracted from this stage. The key from the mapper phase is the individual tokens belonging to a particular reference, and the value is a list of intrinsic metadata such as the reference identifier for the token, the token itself, and the position of token in that reference. With this approach, all the needed information for the rest of the processes is stored in a metadata tag without the use of a shared dictionary as used in the legacy DWM.

For example, let assume we have two references, “A755471,MYRA,AARGAARD-ESPERSEN,1224 MAGNOLIA ST,WINSTON SALEM, NC, 27103, 117-15-8521” and “A944634,IAN,AADLAND,LARS,29021 HIGH SIERRA TRL,SANTA CLARITA,CA,91390,490-46-2048,” and we are to use the tokenizer splitter as the tokenization function. HDWM will take this input data as it is without making data standardization or metadata alignment as done in the traditional Extraction Transformation Load (ETL) process. The splitter tokenization function is then applied on the entity references and the output of the mapper phase is shown in Table 1.

**Table 1:** Output of the tokenization mapper process using splitter tokenizer type.

**Reference**	**Tokenizer mapper output**
A755471,MYRA,AARGAARD-ESPERSEN,1224 MAGNOLIA ST, WINSTON SALEM,NC,27103,117-15-8521	(MYRA, {refID: A755471, tok: MYRA, pos: 1}), (AARGAARD, {refID: A755471, tok: AARGAARD, pos: 2}), (ESPERSEN, {refID: A755471, tok: ESPERSEN, pos: 3}), (1224, {refID: A755471, tok: 1224, pos: 4}), (MAGNOLIA, {refID: A755471, tok: MAGNOLIA, pos: 5}), (ST, {refID: A755471, tok: ST, pos: 6}), (WINSTON, {refID: A755471, tok: WINSTON, pos: 7}), (SALEM, {refID: A755471, tok: SALEM, pos: 8}), (NC, {refID: A755471, tok: NC, pos: 9}), (27103, {refID: A755471, tok: 27103, pos: 10}), (117, {refID: A755471, tok: 117, pos: 11}), (15, {refID: A755471, tok: 15, pos: 12}), (8521, {refID: A755471, tok: 8521, pos: 13})
A944634,IAN,AADLAND,LARS,29021 HIGH SIERRA TRL,SANTA CLARITA,CA,91390,490-46-2048	(IAN, {refID: A944634, tok: IAN, pos: 1}), (AADLAND, {refID: A944634, tok: AADLAND, pos: 2}), (LARS, {refID: A944634, tok: LARS, pos: 3}), (29021, {refID: A944634, tok: 29021, pos: 4}), (HIGH, {refID: A944634, tok: HIGH, pos: 5}), (SIERRA, {refID: A944634, tok: SIERRA, pos: 6}), (TRL, {refID: A944634, tok: TRL, pos: 7}), (SANTA, {refID: A944634, tok: SANTA, pos: 8}), (CLARITA, {refID: A944634, tok: CLARITA, pos: 9}), (CA, {refID: A944634, tok: CA, pos: 10}), (91390, {refID: A944634, tok: 91390, pos: 11}), (490, {refID: A944634, tok: 490, pos: 12}), (46, {refID: A944634, tok: 46, pos: 13}), (2048, {refID: A944634, tok: 2048, pos: 14})

### Frequency generation

**Figure 2:**
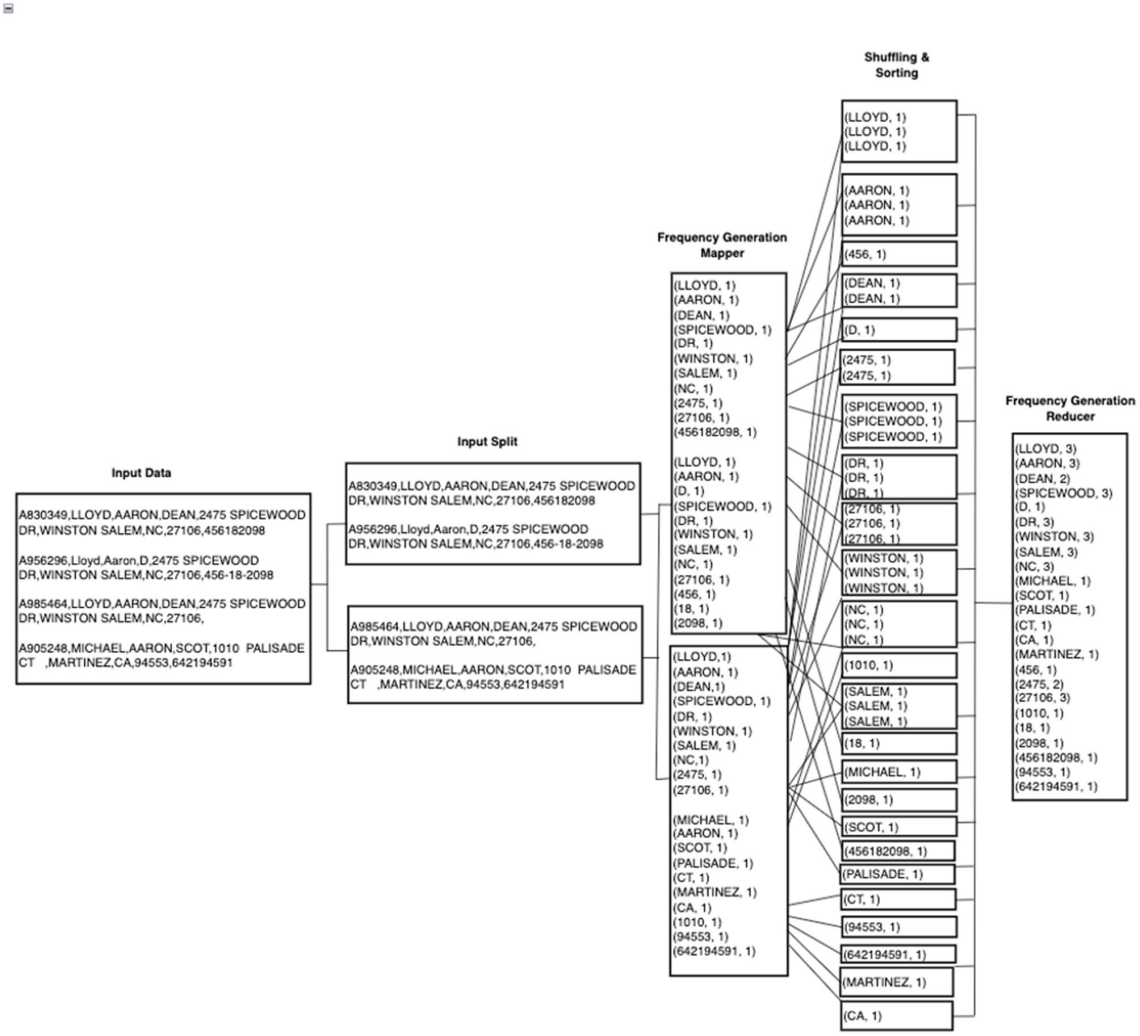
Example of HadoopDWM frequency generation mapper and reducer phases.

The next step is to calculate the frequency of all the tokens across the entire data set. This step is different from the traditional DWM, which creates a shared dictionary of token frequencies. HDWM follows the same concepts of the basic MapReduce word count program to compute token frequency, which is then used in subsequent processes such as Blocking and Stopword removal. In the mapper phase, all tokens are assigned a value of 1 and the reducers shuffle, sorts, and counts how many times a token appears in the given data set. After the mapper, all tokens belonging to a particular key group are sent to the same reducer for the final processing as shown in Figure 2 below.

### Frequency and metadata update

The metadata tag from the tokenization process is updated with the calculated frequency from frequency generation stage. The updated metadata tag contains embedded information that is useful for other processes and is a solution to eliminate the shared dictionary created by the traditional DWM, which results in an “out-of-memory” problem as the volume of data grows exponentially. The updating of the metadata tag is made possible by the merge and join operations in MapReduce. The outputs from the tokenization and frequency generation phases are merged in the mapper phase, and then a join operation in the reducer phase is applied to join the token frequencies to the metadata tag.

For example, Table 2 above is a reducer output that shows updated metadata information where the token “BLVD” has a frequency of 3 and is found in the records “A915661,” “A922259,” and “A992523.” In this example, all three “BLVD” tokens are located at the 6th position in their respective references. Similarly, the token “BRAIN” has a frequency of 2 and is found in the records “A750205” and “A942770.” The position of the token “BRAIN” in both reference is the 1st position. It is to be noted that this is always not the case especially when the dataset has some data quality issues including but not limited to missing values. For instance, if in the reference “A750205,” the last name comes before the first name, the token “BRIAN” will be in the 2nd position whereas if in reference “A942770” first name comes first, the token “BRIAN” will be found in the 1st position as illustrated in Table 2. The positional index of the tokens is an important field that is used to reform the references for string comparison. The output from this stage is the individual token as key and an updated metadata tag, in the form of dictionary key-values, as the value.

**Table 2:** Example of reducer job with updated metadata tag.

**Updated metadata tag key-value pairs**
BLVD | {'refID': 'A915661′, 'pos': 6, 'tok': 'BLVD', 'freq': 3}
BLVD | {'refID': 'A922259′, 'pos': 6, 'tok': 'BLVD', 'freq': 3}
BLVD | {'refID': 'A992523′, 'pos': 6, 'tok': 'BLVD', 'freq': 3}
BRIAN | {'refID': 'A750205′, 'pos': 1, 'tok': 'BRIAN', 'freq': 2}
BRIAN | {'refID': 'A942770′, 'pos': 1, 'tok': 'BRIAN', 'freq': 2}

### Reference reformation

At the reference reformation stage, the token positions are used to group all tokens that belong to a particular reference identifier. This process ensures each token is placed in its right order since they will be used in the similarity comparison stage. The reservation of each token's position will also be used in the cluster evaluation stage where tokens of a reference belonging to a cluster are compared for organization or disorganization using entropy. All blank fields and unwanted characters found in the original reference were eliminated during the tokenization stage and, therefore, are not accounted for when recreating the references back to their original state. One purpose of the reference reformation is to preserve the reference while eliminating the need for a shared dictionary of tokens in the data set as used by the traditional DWM.

Table 3 above is an example of a reference reformation job from HDWM. The input for this MapReduce job was the output from the updated metadata phase. In the mapper phase, the reference identifier for each of the tokens was extracted from the updated metadata tag and that serves as input for the reducer which then aggregates all the tokens and its intrinsic metadata.


(3) $$\begin{array}{l} \left(2:AARONˆ33\right)\to \left(token\;position\;2\;:tokenˆtoken\;frequency\;33\right) \end{array}$$

**Table 3:** Reference reformation with a complete intrinsic metadata information.

A864729 {1: 'REBA^1^', 2: 'AARON^3^3', 3: 'PEDDLE^1^', 4: '516^1^', 5: 'HEATHERTON^1^', 6: 'LN^3^', 7: 'RURAL^1^', 8: 'HALL^1^', 9: 'NC^4^7', 10: '27045^1^'}
A830349 {1: 'LLOYD^4^', 2: 'AARON^3^3', 3: 'DEAN^3^', 4: '2475^6^', 5: 'SPICEWOOD^6^', 6: 'DR^1^2', 7: 'WINSTON^3^1', 8: 'SALEM^3^1', 9: 'NC^4^7', 10: '27106^7^', 11: '456182098^1^'}
A819955 {1: 'JEREMY^2^', 2: 'AARON^3^3', 3: 'TYLER^2^', 4: '3211^4^', 5: 'KINNAMON^4^', 6: 'RD^1^3', 7: 'WINSTON^3^1', 8: 'SALEM^3^1', 9: 'NC^4^7', 10: '27104^1^0', 11: '363197202^1^'}
A812219 {1: 'CELIA^1^', 2: 'AARON^3^3', 3: '1106^1^', 4: 'LASSEN^1^', 5: 'DR^1^2', 6: 'HANFORD^1^', 7: 'CA^3^', 8: '93230^1^', 9: '344^1^', 10: '37^1^', 11: '4232^1^'}
A914099 {1: 'AARON^3^3', 2: 'D^2^', 3: '1117^1^', 4: 'E^2^', 5: 'SEVENTEENTH^1^', 6: 'ST^6^', 7: 'WINSTON^3^1', 8: 'SALEM^3^1', 9: 'NC^4^7', 10: '27105^1^', 11: '636^1^', 12: '32^1^', 13: '8781^1^'}

The key for each reformed reference is the reference identifier, and the value is a dictionary of token position, the token itself, and the token frequency as shown in Table 3. Equation 3 is an example of a token with all its relevant metadata which will be used later in the process. From the equation, the token “AARON” is found at position 2 and has a frequency of 33. Once the references are reformed, they are ready for the blocking phase where blocking keys are extracted from each reference list.

### Blocking

The traditional pairwise approach requires that each reference is compared with all other references. This increases the computational time and causes a lot of workloads for computing resources. To solve this problem and reduce the computational complexity, blocking is used in ER to group references having similar characteristics into the same group before comparing them (Christen, 2012). Blocking is a form of rough matching before the main similarity comparison and linking process in ER and is useful, especially when processing larger volumes of data sets. The blocking process involves three main stages: extraction of blocking tokens from each reference, block key pair creation, and block pair deduplication.

#### Extract blocking tokens

Just as in the legacy DWM, HDWM uses a frequency-based blocking approach where all tokens having a frequency higher than two, up until a given beta threshold, are grouped into the same block for comparison. The first operation in the blocking phase is to extract all tokens that meet a given beta threshold. The token extraction happens in the mapper phase. From each reformed reference, the frequency information is used to only extract and carry along tokens that have a frequency between 2 and the beta value. For instance, if beta is set to 15, all tokens having a frequency from 2 to 15 are preserved and used to create blocking keys. Tokens with frequency of 1 are eliminated due to the assumption that two references that should be compared need to share at least one token. Using the data in Table 3 as an example, to compare reference “A830349” to “A864729,” they should at least have a common token which in this case is “AARON,” and “NC.”

There is also a rule to check the token length, and if the token does not meet the minimum token length that makes a token qualify for blocking, such token is eliminated. Using the data in Table 3 above, and assuming the ‘*minBlkTokenLen*=*5*” all tokens of length < 5 will be excluded as seen in Table 4 below. For instance, as shown in Table 4 below, the tokens “REBA,” “516,” “LN,” “NC” will be removed from the reference “A864729.” Similarly, the tokens “DEAN,” “2,475,” “DR,” and “NC” will all be excluded from the reference “A830349.”

**Table 4:** Mapper output showing remaining tokens after applying “minBlkTokenLen” parameter.

A864729 [1: 'REBA^1^', 2: 'AARON^3^3', 3: 'PEDDLE^1^', 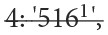 5: 'HEATHERTON^1^', 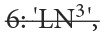 7: 'RURAL^1^', 8: 'HALL^1^' 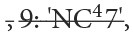 , 10: '27045^1^']
A830349 [1: 'LLOYD^4^', 2: 'AARON^3^3', 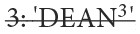 , 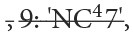 5: 'SPICEWOOD^6^', 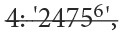 , 7: 'WINSTON^3^1', 8: 'SALEM^3^1', 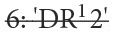 , 10: '27106^7^', 11: '456182098^1^']
A819955 [1: 'JEREMY^2^', 2: 'AARON^3^3', 3: 'TYLER^2^', 4: '3211^4^', 5: 'KINNAMON^4^', 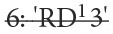 , 7: 'WINSTON^3^1', 8: 'SALEM^3^1', 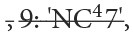 , 10: '27104^1^0', 11: '363197202^1^']
A812219 [1: 'CELIA^1^', 2: 'AARON^3^3', 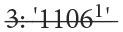 4: 'LASSEN^1^', 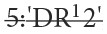 , 6: 'HANFORD^1^', 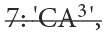 8: '93230^1^', 9: '344^1^', 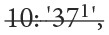 11: '4232^1^']
A914099 {1: 'AARON^3^3', 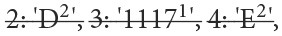 , 5: 'SEVENTEENTH^1^', 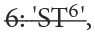 , 7: 'WINSTON^3^1', 8: 'SALEM^3^1', 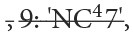 , 10: '27105^1^', 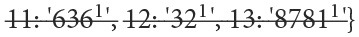 }

Another rule in the blocking phase is to decide whether to exclude numeric tokens from forming blocking keys or maintaining them. Again, using the reformed references in Table 3 as an example, if numeric tokens are to be excluded, denoted by the parameter “*excludeNumericTokens*,” the token “27,045” will be removed from the “A864729” reference, the tokens “27,106” and “456,182,098” will be excluded from the “A830349” reference, the tokens “27,104” and “363,197,202” will be removed from the “A819955” reference, and finally, the tokens “93,230,” “344,” and “4,232” will be removed from the “A812219” reference. Column 3 of Table 5 shows the final mapper output after applying “minBlkTokenLen” and “excludeNumericTokens” parameters and excluding all tokens having frequency of 1. For instance, “REBA,” “PEDDLE,” “516,” “HEATHERTON,” “RURAL,” “HALL,” and “27,045” were all removed from the “A864729” reference because they all have frequency of 1.

**Table 5:** Mapper output showing remainder tokens after applying “excludeNumericTokens.”

**Ref. ID**	**Tokens after applying “excludeNumericTokens” parameter**	**Tokens left for block creation**
A864729	[2: 'AARON^3^3', 3: 'PEDDLE^1^', 5: 'HEATHERTON^1^', 7: 'RURAL^1^', 8: 'HALL^1^', 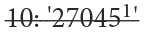 ]	[AARON']
A830349	[1: 'LLOYD^4^', 2: 'AARON^3^3', 5: 'SPICEWOOD^6^', 7: 'WINSTON^3^1', 8: 'SALEM^3^1', 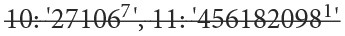 ]	['LLOYD, AARON, SPICEWOOD, WINSTON, SALEM]
A819955	[1: 'JEREMY^2^', 2: 'AARON^3^3', 3: 'TYLER^2^', 4: '3211^4^', 5: 'KINNAMON^4^', 7: 'WINSTON^3^1', 8: 'SALEM^3^1', 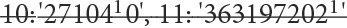 ]	[JEREMY, AARON, TYLER, KINNAMON, WINSTON, SALEM]
A812219	[1: 'CELIA^1^', 2: 'AARON^3^3', 4: 'LASSEN^1^', 6: 'HANFORD^1^', 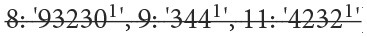 ]	[AARON]
A914099	[1: 'AARON^3^3', 5: 'SEVENTEENTH^1^', 7: 'WINSTON^3^1', 8: 'SALEM^3^1', 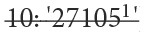 ]	[AARON, WINSTON, SALEM]

#### Creation of blocking keys

HDWM uses the block-by-pair approach where a pair of references will be compared only if they have at least two tokens in common as discussed earlier. The tokens that were preserved now are used as blocking keys. The keys can either be pairs of keys, if using “blockByPairs” is set to true or single keys if “blockByPairs” is set to false. For instance, if the reserved tokens in a reference were “[BRIAN, ABADIR, and PINE]” and block-by-pairs is true, the keys “BRAINABADIR, BRIANPINE, and ABADIRPINE” will be created as blocking keys for such reference. On the other hand, if block-by-singles is set to true, the keys “BRIAN,” “ABADIR,” and “PINE” will be formed as blocking keys. In the reducer, all reference identifiers having the same blocking key are grouped.

**Figure 3:**
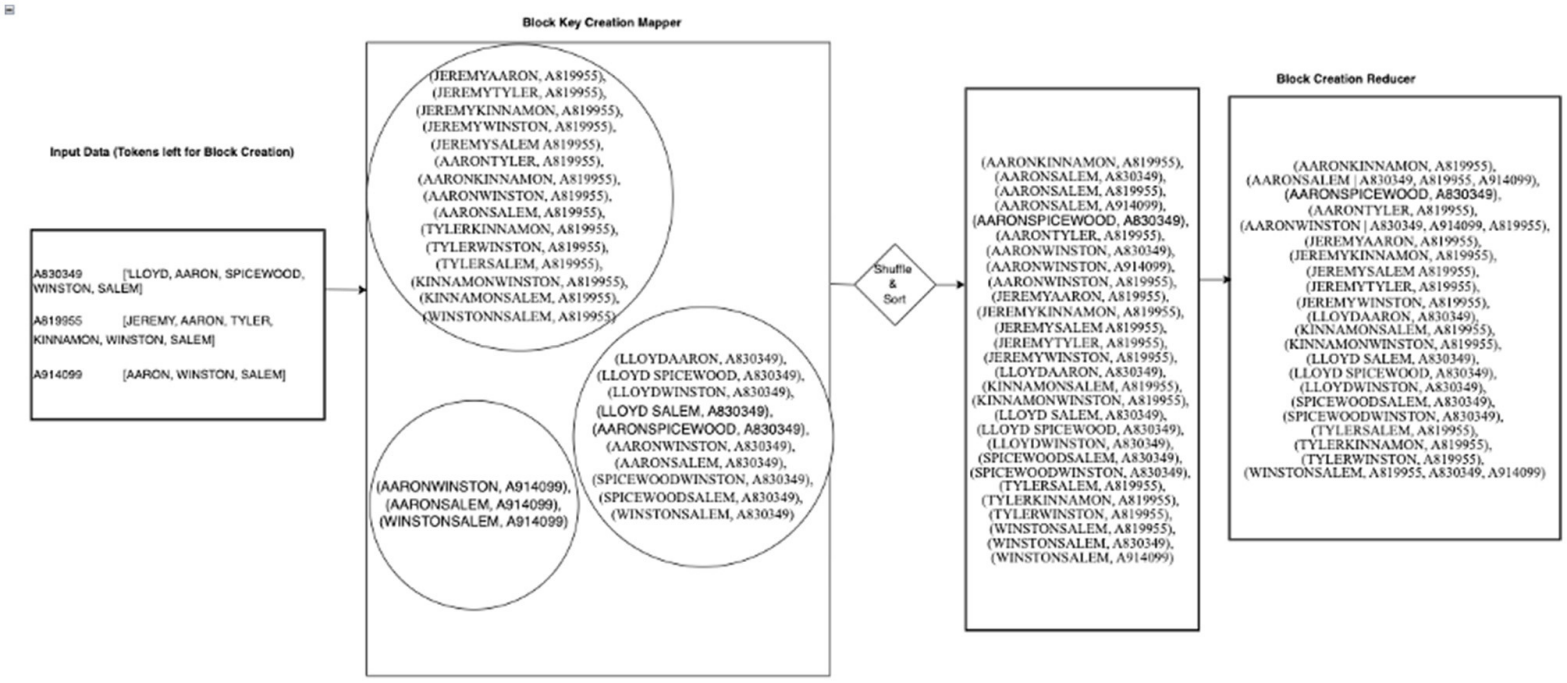
Working example of block creation job in HDWM.

Figure 3 shows a working example of the blocking key creation using the remainder of tokens after removing all unwanted tokens. The input of this job is the output from the third column of Table 5 above. It can be noted that all references having single tokens (reference “A864729” and “A812219”) were eliminated and not used to create blocking keys. This is because of the “block-by-pair” logic explained above. Since the creation of blocking keys need at least two tokens in each reference, all references with singular tokens are disqualified.

#### Blocking pair deduplication

Since a particular blocking key may be found in more than one reference, there is a higher chance of having pairs of references that will be brought together by that key. In the block deduplication phase, all pairs are processed in a reducer to eliminate all duplicates before sending the final pairs to the similarity comparison function for linking and matching. This step is crucial to prevent the possibility of comparing the same pair of references more than once, which will add to the computational complexity. In the mapper phase of the block deduplication, pairs of reference identifiers from each block key are created. The pairs are sorted in ascending order of magnitude, and these are the pairs that will end up in the reducer. In the reducer, all duplicate reference identifier pairs are eliminated. This is because it is not efficient to compare the same pair of reference more than once. Comparing the same pair of reference more than once increase the computational time in the similarity comparison phase. For instance, if the pairs “(A830349, A914099),” “(A819955, A839349),” and “(A819955, A914099)” were created by the keys, “AARONWINSTON” and “WINSTONSALEM” and the same pairs had already been created by the key “AARONSALEM,” the reducer will emit only unique pair from each key group.

### Similarity comparison and linking

Sigma is a parameter for removing stopwords and should always be higher than beta. Stopwords are tokens that have a frequency higher than a given sigma value (Al Sarkhi and Talburt, 2018, 2019). Prior to comparing a pair of references for similarity, stopwords are removed from each pair of references using a sigma threshold. This process is performed to reduce the number of tokens that are left in a reference to be compared. This helps facilitate the matching process and computational time. For instance, if a sigma threshold is set to 45, all tokens with a frequency higher than 45 are eliminated from the reference, and the remaining tokens go through a matrix comparator program to determine whether there is a match or no match. HDWM uses a variant of the Monge Elkan scoring matrix, which is created for the DWM (Li et al., 2018). The scoring matrix compares two tokens using a Mu threshold between the values of 0 and 1, where the higher the value, the higher the similarity. If references have a similarity score equal to or higher than the set Mu threshold, such references are said to be equivalent to each other and are, therefore, a match and vice versa. This process only happens in a reducer, and the output is a list of linked pairs of references. At the end of each iteration, the Mu value is increased by a Mu-iterate value between 0 and 1 for the next iteration. If a Mu reaches more than 100%, the program ends and produces a linked index file containing linked pairs.

Table 6 shows an output from the similarity comparison job in HDWM given a linking threshold (mu) of 90%. The third column of the table shows the linking decision made by HDWM as to whether the pair was linked or not linked. Per the given threshold for linking pairs, the “A905248.A950657,” “A739417.A762973,” and “A756280.A865919” pairs did not match and therefore were eliminated from the output. The final out of the similarity comparison is a combination of three items for each pair. The program outputs the linked pair, the inverse of the linked pair and the pair itself using only the key for the pair. The key of the output is a composite key containing the left reference identifier and right reference identifier. The value of each pair is the second item if the composite key as shown in equation 4.

**Table 6:** Comparing similarity score to a 0.90 mu threshold to determine match or no match.

**Input data (deduplicated block pairs)**	**Similarity score**	**Linking decision**	**Output (linked pair, inverse pair, pair key self)**
A819955.A975276	0.99	Match	A819955.A975276, A975276 A975276.A819955, A819955 A819955.A819955, A819955
A824044.A935026	1.0	Match	A824044.A935026, A935026 A935026.A824044, A824044 A824044.A824044, A824044
A824917.A875214	0.92	Match	A824917.A875214, A875214 A875214.A824917, A824917 A824917.A824917, A824917
A830349.A956296	1.0	Match	A830349.A956296, A956296 A956296.A830349, A830349 A830349.A830349, A830349
A830349.A956423	0.99	Match	A830349.A956423, A956423 A956423.A830349, A830349 A830349.A830349, A830349
A905248.A950657	0.72	No-match	
A739417.A762973	0.89	No-match	
A756280.A865919	0.74	No-match	

### Transitive closure

HDWM implements transitive closure in a reducer phase, which is a reference level linking to form clusters. An identity mapper is used in this stage to shuffle and sort each input for the next transitive closure iteration. The logic of transitive closure states that if reference 1 and reference 2 are equivalent to each other, and reference 2 is equivalent to reference 3, then references 1, 2, and 3 are all equivalent to each other and, therefore, form a cluster. HDWM uses the Connected Components with MapReduce (CCMR) algorithm, which was originally proposed by Seidl et al. (2012) and later improved by Kolb et al. (2014). CCMR searches through links of references to find all connected components to a particular reference, forming a star-like node and edge graph of clustered references. In each transitive closure iteration, the merge state and the local max state are recorded using custom MapReduce counters. These counters are used to decide whether to execute the next iteration of transitive closure. Pairs in a merge state are continuously chained to recreate new pairs until a fully local max state is obtained. The transitive closure iteration ends if there are no key groups that were found in a merged state, and therefore, the merge state counter is 0.

The input of the CCMR algorithm is the output from the similarity comparison phase. Table 7 shows an iteration of the transitive closure algorithm. In the mapper phase, an identity mapper is utilized where no computations are made. The output from the mapper is sorted and then fed into the reducer which then uses the logic in the CCMR algorithm to find connected components of each key group as seen in Equation 4 below. Equation 4 represent a group of references that will serve as input for the CCMR transitive closure algorithm. The reference identifiers colored in blue represent composite key with the first item of the composite key being the group key. The reference identifiers color coded orange represents all the values for that group.


(4) $$\begin{array}{l} A819955.A819955\;A819955\\ A819955.A975276\;A975276 \end{array}$$

**Table 7:** Example of transitive closure output using CMR algorithm.

**Input data**	**Sorted linked pairs**	**Transitive closure iteration 1**
A819955.A975276 A975276 A975276.A819955 A819955 A819955.A819955 A819955 A824044.A935026 A935026 A935026.A824044 A824044 A824044.A824044 A824044 A824917.A875214 A875214 A875214.A824917 A824917 A824917.A824917 A824917 A830349.A956296 A956296 A956296.A830349 A830349 A830349.A830349 A830349 A830349.A956423 A956423 A956423.A830349 A830349 A830349.A830349 A830349	A819955.A819955 A819955 A819955.A975276 A975276 A824044.A824044 A824044 A824044.A935026 A935026 A824917.A824917 A824917 A824917.A875214 A875214 A830349.A830349 A830349 A830349.A830349 A830349 A830349.A956296 A956296 A830349.A956423 A956423 A875214.A824917 A824917 A935026.A824044 A824044 A956296.A830349 A830349 A956423.A830349 A830349 A975276.A819955 A819955	A819955.A819955 A819955 A819955.A975276 A975276 A824044.A824044 A824044 A824044.A935026 A935026 A824917.A824917 A824917 A824917.A875214 A875214 A830349.A830349 A830349 A830349.A956296 A956296 A830349.A956423 A956423

### Cluster evaluation

Clusters formed at the transitive closure stage are evaluated to determine organization or disorganization between clusters using Shannon Entropy. An epsilon threshold between 0 and 1 is used at this stage, where 0 means higher disorganization and 1 means higher organization. Although a reference may end up in a particular cluster, it may not necessarily mean such cluster has all the references that are equivalent to each other. The cluster evaluation produces two main categories of outputs: a good cluster of references and a bad cluster of references. If all the clusters are good, then the process will end, and if vice versa, all bad clusters are selected for reprocessing in the next phase. At the end of each phase, the epsilon is increased using an epsilon-iterate value, and that serves as the new epsilon for the next iteration. The evaluation process happens in a reducer phase. An identity mapper is utilized to shuffle and sort the input of this phase to group all clusters and ready for the reducer. Clustered references that successfully goes through the cluster evaluation phase are tagged as “usedRefs” in order not to reprocess them in the next program iteration.

### ER evaluation matrix

At the end of the process, if no further computation is to be made, the final good clusters are collated and used to calculate the performance of the system. The matrix is calculated only if a truth set file is given by the end user. A truth set is a file that contains the correct matches of each reference and the cluster it belongs. It contains only two columns where the first column is the reference identifier, and the second column is the cluster identifier. If a truth file is given, it is hidden from the ER process and only utilized to evaluate the system's performance. The creation of a truth file is a complete process and so not always available. A pairwise comparison approach is adopted in ER in which the goal is to compare two references for equivalence. These pairs are counted using the function in Equation 5 below.


(5) $$\begin{array}{l} pairs=\;\left(n*\left(n-1\right)\right)/2 \end{array}$$

The matrix calculated in this process are shown in Equations (6–8) below. Linked pairs are pairs that were marked as equivalent from the HDWM system, equivalent pairs are pairs that are supposed to be referring to the same real-world objects, and true pairs are the intersection between equivalent pairs and linked pairs. Precision calculates the ratio of all the good links to the total number of links that were made during the process. The recall, on the other hand, measures all the pairs that are belong to the correct clusters and are equivalent. It looks at the universe of equivalent pairs. While precision ensure that created clusters contain only references that are equivalent to each other, recall ensures all references to a given entity end up in the same cluster. F-measure is used to balance the precision and recall and is the harmonic mean of the precision and recall.


(6) $$\begin{array}{l} Precision=TP/L \end{array}$$

(7) $$\begin{array}{l} Recall=TP/E \end{array}$$

(8) $$\begin{array}{l} F-measure=\left(2*P*R\right)/\left(P+R\right) \end{array}$$

## Experiment and results

### Dataset

Two separate sets of data were used for experimentation, including a generated name-address samples used as base benchmark data for the legacy DWM. The data set used was generated using the SOG system (Talburt et al., 2009) comprising of names and addresses as shown in **Table 9**. The data ranges from poor-quality formats to good-quality formats and single layouts to mixed layouts. The datasets ending with the letters “G” and “P” are good quality and poor quality, respectively. On the other hand, datasets ending with the letters “GX” and “PX” are the good-mixed quality layout and poor-mixed quality layout, respectively. The good-quality data sets have the headers: recID, fname, lname, mname, address, city, state, zip, and ssn. Whereas, the poor-quality data sets have the headers: RecID, Name, Address, City State Zip, PO Box, POCity State Zip, SSN, and DOB. Both systems are given the same parameter file containing settings to be used in the execution.

The second set of data are publicly available datasets used by several researchers to evaluate ER system's performance (Köpcke et al., 2010). Four separate publicly available datasets namely “Affiliations,” “Geographic Settlements,” “Music-Brainz,” and “NC Voters” were used to test the scalability of HDWM. The North Carolina voter's data set containing 5 million references with ~3.5 million clusters were analyzed and used for this research. Two additional variants of the NC Voters dataset were created including a 7 million version and a 50 million version using a reference duplication algorithm by copying the same references from the 5 million file and assigning unique identifiers to the copied references. This was used to perform volume experimentation and versatility of HDWM. The descriptions of the publicly available datasets are shown in Table 8 below.

**Table 8:** Publicly available datasets used to test scalability of HDWM.

**Sample**	**Size (entities)**	**Description**
Affiliations	2,260	Contains database affiliation strings. This includes address of institutions.
Geographic Settlements	3,054	Contains real-world entities from freebase, geonames, NYTimes, and DBpedia.
Music Brainz 200 k	193,750	Contains songs from a music database and duplicates are generated for research purposes
NC-Voters 5 million	5,000,000	Contains real-world records from North Carolina voter's registry
NC-Voters 7 million	7,000,000	Variation of NC-Voters-5 million
NC-Voters 50 million	50,000,000	Variation of NC-Voters-5 million

### Benchmarking HDWM with legacy DWM

This section shows the experimentation of HDWM with 18 generated data files, which are also used to test the performance of the legacy DWM. Given sizes these 18 samples were less than the default HDFS block size of 128 MB, a single-node Hadoop cluster was utilized to prove that it achieves the same clustering result as the legacy DWM. The workstation used is a 64-bit Ubuntu 23.04 server with a stable Hadoop 3.3.1 and OpenJDK 8 installed. The server is equipped with a 4-core i3 Intel 4th generation CPU at 3.10 GHz base speed and 8GB DDR3 RAM. Some statistics recorded from the experiment include the number of Linked Pairs, True Pairs, Equivalent Pairs, Precision, Recall, and F-measure, as shown in Table 9.

**Table 9:** Comparison of HDWM and legacy DWM.

**Sample**	**Refs read**	**Quality**	**Beta**	**Mu**	**Sigma**	**Epsilon**	**System**	**True pairs**	**Expected pairs**	**Linked pairs**	**Precision**	**Recall**	***F*-measure**
S1G	50	Good	6	0.6	7	0.23	LDWM	27	27	27	1.0	1.0	1.0
							HDWM	27	27	27	1.0	1.0	1.0
S2G	100	Good	12	0.72	14	0.29	LDWM	48	48	52	0.9231	1.0	0.96
							HDWM	48	48	52	0.9231	1.0	0.96
S3Rest	868	Good	4	0.67	50	0.44	LDWM	101	112	104	0.9712	0.9018	0.9352
							HDWM	101	112	104	0.9712	0.9018	0.9352
S4G	1,912	Good	8	0.74	51	0.31	LDWM	906	990	939	0.9649	0.9152	0.9394
							HDWM	906	990	939	0.9649	0.9152	0.9394
S5G	3,004	Good	18	0.76	66	0.25	LDWM	1,395	1,526	1,462	0.9542	0.9142	0.9338
							HDWM	1,395	1,526	1,462	0.9542	0.9142	0.9338
S6GeCo	19,998	Good	125	0.77	850	0.41	LDWM	22,696	23,232	23,628	0.9606	0.9769	0.9687
							HDWM	22,696	23,232	23,628	0.9606	0.9769	0.9687
S7GX	2,912	Good	15	0.76	41	0.29	LDWM	1,331	1,468	1,408	0.9453	0.9067	0.9256
							HDWM	1,331	1,468	1,408	0.9453	0.9067	0.9256
S8P	1,000	Poor	23	0.67	32	0.13	LDWM	1,877	2,811	2,211	0.8489	0.6677	0.7475
							HDWM	1,877	2,811	2,211	0.8489	0.6677	0.7475
S9P	1,000	Poor	32	0.72	33	0.15	LDWM	1,963	2,855	2,290	0.8572	0.6876	0.7631
							HDWM	1,963	2,855	2,290	0.8572	0.6876	0.7631
S10PX	2,000	Poor	45	0.73	61	0.06	LDWM	8,132	11,878	9,194	0.8845	0.6846	0.7718
							HDWM	8,132	11,878	9,194	0.8845	0.6846	0.7718
S11PX	3,999	Poor	108	0.72	109	0.15	LDWM	15,921	23,456	19,678	0.8091	0.6788	0.7382
							HDWM	15,921	23,456	19,678	0.8091	0.6788	0.7382
S12PX	6,000	Poor	67	0.71	70	0.15	LDWM	21,734	31,735	24,867	0.874	0.6849	0.768
							HDWM	21,734	31,735	24,867	0.874	0.6849	0.768
S13GX	2,000	Good	40	0.79	66	0.22	LDWM	1,750	1,949	1,979	0.8843	0.8979	0.891
							HDWM	1,750	1,949	1,979	0.8843	0.8979	0.891
S14GX	5,000	Good	94	0.81	160	0.1	LDWM	4,245	4,865	4,621	0.9186	0.8726	0.895
							HDWM	4,245	4,865	4,621	0.9186	0.8726	0.895
S15GX	10,000	Good	160	0.84	225	0.38	LDWM	8,447	9,727	9,148	0.9234	0.8684	0.8951
							HDWM	8,447	9,727	9,148	0.9234	0.8684	0.8951
S16PX	2,000	Poor	48	0.73	64	0.06	LDWM	8,275	11,986	9,343	0.8857	0.6904	0.7759
							HDWM	8,275	11,986	9,343	0.8857	0.6904	0.7759
S17PX	5,000	Poor	91	0.73	95	0.15	LDWM	19,643	29,042	22,580	0.8699	0.6764	0.761
							HDWM	19,643	29,042	22,580	0.8699	0.6764	0.761
S18PX	10,000	Poor	92	0.73	116	0.12	LDWM	37,676	57,004	44,421	0.8482	0.6609	0.7429
							HDWM	37,676	57,004	44,421	0.8482	0.6609	0.7429

The result from Table 9 above shows that HDWM can cluster equivalent references when given optimal parameters. The sample with the highest number of references is S6GeCo having 19,998 references. With optimal parameters by HDWM, we were able to achieve a precision of 0.9606, recall of 0.9769, and f-measure of 0.9687. This means out of the pairs the system linked were true links and were referring to the same entities as predicted by the system.

#### Volume (scalability) test

The scalability of HDWM was tested in a fully distributed cluster using computational nodes from the Arkansas High-Performance Computing Center's Pinnacle HPC. The workstation server is equipped with two intel Broadwell processors with two total cores and two threads. A total of 20 compute nodes were loaded to run the 5 million records of size 187.44 MB, 30 compute nodes to run ~7 million reference, and 50 nodes to run the 50 million records of size 1.83 GB. Each compute node is equipped with Intel(R) Xeon(R) Gold 6130 CPU^@^2.10 GHz base speed, with 32 cores, and a memory of 192 GB.

Some statistics extracted from the tokenization phase, blocking phase, linking phase, and cluster evaluation phase using S6GeCo, and NC Voters are shown in Table 10 below. There are four main conditions under which the program iteration will exit. If the blocked pair list is empty, if the linked pair list is empty, if the transitive closure cluster list is empty, or if the new Mu value is >1.0, the program exit code will be triggered to end the execution.

**Table 10:** Program statistics for up to 50 million records.

**Sample**	**Iteration**	**Tokens found**	**Numeric tokens**	**Block keys created**	**Pairs created by block keys**	**Unduplicated block pairs**	**Linked pairs**	**Total clusters**	**Refs in clusters**
S6-GeCo	1	292,928	148,626	630,818	312,353	69,924	16,645	3,319	13,246
	2			246,448	13,859	9,751	0		
NC-Voters-5 mil	1	20,965,282	4,909,829	125,678	6,329	6,321	4,947	3,956	8,414
	2			122,791	4,673	4,671	3,295	2,595	5,606
	3			122,791	4,673	4,671	3,289	2,590	5,596
	4			122,791	4,673	4,671	3,287	2,590	5,596
NC-Voters-7 mil	1	29,357,678	6,956,318	2,121,898	22,844,681	22,585,957	1,065,973	836,693	1,754,634
NC-Voters-50 mil	1	209,652,820	49,098,290	879,287	4,729,786	4,600,726	4,597,210	82,378	838,822
	2			8,982	1	1	0		

Table 11 above shows a comparative analysis of HDWM and Famer entity clustering system proposed by Saeedi et al. (2017). The table also show that for the Affiliations, Geographic Settlements, and Music Brainz datasets, HDWM outperforms Famer in terms of total number of references clustered. We believe the over clustering in HDWM is due to the use of optimal parameters used by the system. These parameters include by not limited to “epsilon” which is used to calculate the quality of a cluster after linking. Famer uses connected component graph approach for clustering linked references.

**Table 11:** Cluster statistics and ER matrix for real-world datasets.

**Sample**	**Size**	**System**	**# of linked pairs**	**# of clusters**
Affiliations	2, 260	HDWM	26,844	814
		Famer	32,816	330
Geographic settlements	3, 054	HDWM	4,501	1,073
		Famer	4,391	820
Music Brainz 200 k	193, 750	HDWM	346,914	111,901
		Famer	162,500	100,000
North Carolina Voters 5 M	5, 000, 000	HDWM	1,650	3,496,553
		Famer	331,384	3,500,840

#### Shuffling behavior of HDWM

**Figure 4:**
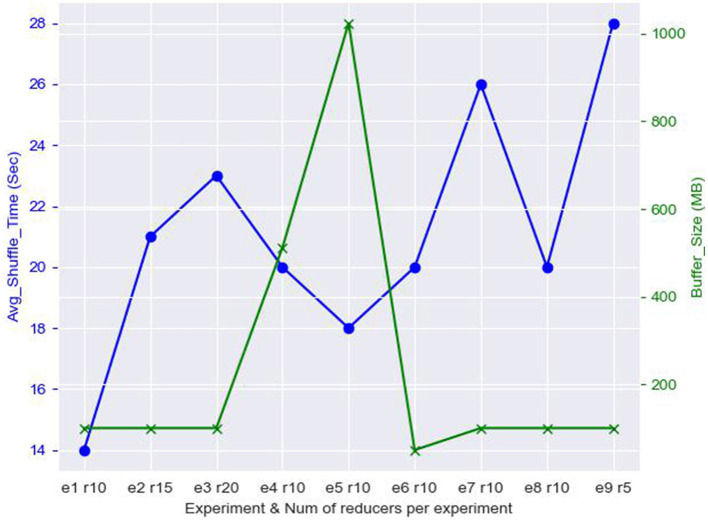
Analysis of reducer count, buffer size, and shuffle time of HDWM block pair deduplication.

In this section, we demonstrate the behavior of the shuffling stage of HDWM. Since HDWM si an iterative system with many phases, we isolated one of the data-intensive phases of the process, Block Pair Deduplication, and showed how data copied from the mapper phase are shuffled before being fed into available reducers on the cluster. There are multiple optimization techniques, however, we focused on the number of reducers and the buffer size used by reducers for sorting and shuffling intermediate data from the mapper's output. The results of HDWM's shuffling in the Block Pair Deduplication phase using North Carolina voter's dataset has been presented in Figure 4 below. The default buffer size in HDFS is 100 MB, the data size that needed to be shuffled in this process was 5,027.30 MB.

From Figure 4, it can be observed that reducing the number of reducers increases the amount of time used to shuffle the data. For instance, when the number of reducers was reduced from 10 to 5 as shown in experiment 9 (e9 r5), the average shuffle time, in seconds, was increased from 20 s in experiment 8 (e8 r10) to 28 s (e9 r5). On the other hand, increasing the buffer size in experiment 4 (e4 r10) from 512 MB to 1 GB in experiment 5 (e5 r10), there was a slight reduction in the average shuffle time taken by the reducers. Although increasing the number of reducers per job decreases the computational time, it impacts the shuffle time since lots of records need to be merged, sorted, and shuffled before the reducer phase. There is I/O overhead cost when the mappers emit more data which need to be merged and ensure that each reducer receives the intermediate output for processing. Again, increasing the buffer size in HDWM for the reducers decreases the time taken to shuffle intermediate outputs from the mappers. This is because the amount of memory for each shuffling operation. This works best especially for I/O intensive mapreduce jobs.

## Conclusion and future work

With the exponential growth of data in the era of big data, the DWM is unable to meet the standard of clustering larger datasets. Although the DWM can successfully cluster equivalent references, it is only able to do so to just a few 1,000 references, making it unscalable. This work focused on developing a scalable version of an unsupervised data curation engine using Hadoop MapReduce. We tackled the issue of single-threaded design of the DWM by using the parallel capability of MapReduce. We also solve the problem of shared single memory by distributing the workload onto multiple computational nodes.

The hadoop-based design of the DWM can cluster both good and poor-quality data sets using a set of parameters optimally selected by a Parameter Discovery Process. HDWM was experimented with 18 generated names and addresses, and a benchmark result shows both DWM and HDWM achieve the same cluster results. The scalability of the system was proved via experimentation with up to 50 million records publicly available data set. We also demonstrated the capability of the proposed system to cluster datasets of varying formats. From our experiments, we conclude that HDWM works best in terms of precision, recall, and f-measure when it is given optimal system parameters. This is evident when HDWM was experimented using the benchmark datasets which were used for the legacy DWM. These datasets have optimal staring parameters which provide the best possible clustering results. This is possible by inculcating a Parameter Discovery Process proposed by Anderson et al. (2023) into a distributed environment to be used by the HDWM system. At each iteration of the HDWM system, statistics such as unique tokens, numeric token, etc. which are used by the PDP can be extracted to predict the next best possible value.

Future work will focus on adopting load-balancing techniques to ensure an equal amount of data is processed by each reducer. We will improve the parallelism of the reducers for all jobs by adjusting the number of reducer tasks to suite our system. We believe adopting load-balancing techniques will significantly decrease the computational time at the reducer phases. We also intend to explore the performance of HDWM using poor quality data sets in mixed format. Another area of research is to explore the performance of HDWM in other domains, such as healthcare records.

Although the adoption of load-balancing techniques will help reduce the computational time, it can further be improved when a more efficient data processing technique is used. MapReduce reads data from disk and then writes the intermediate results of the mapper tasks back to disk. The read and write operation in MapReduce causes overhead cost. In future work, we will focus on refactoring HDWM into PySpark for even faster data processing thereby improving the total execution.

We analyzed the shuffle performance of HDWM and conclude that there is an improvement in the time used by reducers for shuffling the intermediate outputs from mappers when number of reducers is fine-tuned with the buffer size memory used for shuffling. Although there are numerous shuffle optimization techniques in MapReduce, only buffer size and tuning the level of parallelism via number of reducers were explored. We also will improve the shuffling and sorting processes for all the steps presented in this research in future work. This include analyzing the relationship between CPU cores and the shuffle time, reducing the number of record spilled to disk, and also using “shuffle parallel copies” configuration in MapReduce.

## Data availability statement

Publicly available datasets were analyzed in this study. This data can be found at: North Carolina Voters 5m (https://dbs.uni-leipzig.de/research/projects/object_matching/benchmark_datasets_for_entity_resolution).

## Author contributions

NH: Conceptualization, Methodology, Software, Visualization, Writing—original draft. JT: Funding acquisition, Resources, Supervision, Writing—original draft, Methodology. KA: Data curation, Writing—original draft. DH: Writing—review & editing.

## Footnotes

^1^“Apache Hadoop.” https://hadoop.apache.org/ (accessed June 02, 2023).

^2^ https://bitbucket.org/oysterer/hadoop-dwm/src/master/
